# Supplementary material for: Occupational radiation exposure dose and associated factors among radiology personnel in Eastern Amhara, Ethiopia
Source: PLoS One. 2023 May 25;18(5):e0286400. doi: 10.1371/journal.pone.0286400 (PMC10212102; doi:10.1371/journal.pone.0286400)
Supplement: Attachment — Submitted filename: Resopnse to Reviewers.doc [file pone.0286400.s002.doc]

**Response to academic editor**

Firstly, we would like to thank the academic editor for facilitating the process. We authors tried to address all your concerns such as the study rationale, references, appropriate dose definitions, and description of the study design. Please see the responses given for reviewers and the revised manuscript.

**Response to reviewers’ comments**

We are grateful to reviewers for your valuable comments and questions that are helpful for improving the quality of the manuscript. The responses for each point are done as follows.

**Reviewer #1:**

- Line 37 (Abstract): …those with a p-value < 0.5 were judged significant.
  I thought you wanted to write “<0.05” instead of “<0.5”

**Response:** Yes, thank you. That’s editorial error. (Please see the revised version).

- Lines 47-48: “effective medical exposure of the world population”
  It would be better to use technical terms such as effective dose correctly. I think you mean “global average of annual per caput effective dose due to medical exposure”.

**Response: Yes, thank you. It is corrected accordingly.**

- Lines 159-161: The calculated mean (± SD) annual TLD readings…as occupational radiation exposure dose.

**Response: The individual radiation doses were regularly read and recorded by ERPA and DosiMed testing laboratory, which are the only two organizations giving this service in Ethiopia currently. By document review (secondary data) the annual doses of radiology personnel (TLD readings) were extracted from the databases of ERPA and DosiMed testing laboratory. Then, mean (± SD) were calculated.**

- Line 186: Document review was used to extract the annual Thermoluminescent Dosimeter (TLD) readings…

I think short descriptions related to quality assurance of TLD measurement should be added. For example, TLD calibration procedures, types of TLD reading devices, and possibility of improper use of TLD are important for the quality assurance, I think.

**Response:** **The annual TLD readings were taken from the ERPA and DosiMed testing laboratory (secondary data) for this study. Computerized Vinten Solaro machine was used by the two organizations as a TLD reader. The quality assurance of each TLD machine was done regularly by the experts in the two organizations. Furthermore, each radiology personnel were equipped with two TLDs (when one is sent for reading, the other is used). The radiology personnel were responsible to declare the TLD they use every two months by the Authority. So, the possibilities of improper use of TLD were very unlikely.** **(Please see the revised version)**

- Line 167: Collective dose: the sum of all individual effective doses
  How to calculate “individual effective doses” should be described. I think these were calculated from “deep dose” and “shallow dose”. When protective equipment such as lead apron is used, attention should be paid for calculating effective doses from measured doses, as described in the following references.
  Evaluation of the effectiveness of X-ray protective aprons in experimental and practical fields. Radiol Phys Technol (2014) 7:158–166, DOI 10.1007/s12194-013-0246-x
  Effective dose to staff from interventional procedures: estimations from single and double dosimetry. Radiat Prot Dosim (2009), Vol. 136, No. 2, pp. 95–100
  doi:10.1093/rpd/ncp155

**Response:** **The individual effective dose was calculated from the deep dose (HP10) and shallow dose (HP 0.007), which indicates the personal dose at a tissue depth of 10 mm and 0.007 cm. Using the lead apron, does not affect the individual effective dose as the TLD is put under the apron. (Please see the revised version)**

- Line 172: Ref [29] and Line 174: Ref [43]
  I think these ref Nos. are wrong. Reference citation before Line 172 can be found in Line 139, which referred to No. [23].

**Response: Thank you. Ref [29] is changed to ref [24] and Ref [43] is changed to Ref [25]. All the references after Ref [23] are corrected accordingly.**

- Line 252: According to the text, lead apron, eye goggle, thyroid collar, and lead glove were used in some cases. In the case of using the lead apron, was TLD worn under the apron? If TLD was worn over the apron, shielding effects should be considered in estimating deep dose, shallow dose and effective dose, I think.

**Response: Thank you, it is a great concern. The TLD were worn under the lead apron. This is highly recommended by ERPA, the governmental authority in Ethiopia, and the radiology personnel were aware of that.**

- Lines 270-272, 275-286, 291, 292, 350-352, 364 and 368: effective deep and shallow doses.
  What are the definitions of “effective deep and shallow doses”? Are these different from “deep dose” and “shallow dose”? It should be clarified.

**Response:** **in case of TLD measurements and for our study, no difference. Effective deep dose or simply deep dose is a whole-body dose. Shallow dose or effective shallow dose (a shallow dose equivalent to the whole body) is a skin dose.**

- Lines 295-296: Deep and shallow doses were compared with effective dose (0.6 mSv). I think the definitions of these doses are different from each other. In particular, these dose values could be different even in the same exposure condition, when protective equipment was used (see comments on Line 167).

Also, studies from different countries were compared in Lines 303-312. It should be confirmed if these comparisons were done in terms of the same type of dose (e.g., effective dose).

**Response:** **It is to mean** **the annual effective deep and shallow dose. It is corrected in the revised version.**

- Table 3 (lines 262-265): More explanation is needed for items in “Leakage measurement” and “Collimator and beam alignment”, I think. In the text, explanation on Table 3 is too short (lines 262-265).
  For example, the maximum dose for “work area dose result, door to the toilet” was 28 mSv. Is this within acceptable range?

**Response:**

- **All the quality control test results were taken from ERPA data base by document review, which was done by the experts of ERPA. Leakage measurement {a measure of leakage radiation from the radiation source assembly (secondary radiation)} was measured at the collimator and at the tube. It is deviated acceptably by 0.24% both at collimator and at the tube.**
- **Collimator and beam alignment: the beam alignment was deviated from the long axis of the collimator (long axis variance) from -1.25% to +1.5% and from the short axis of the collimator (short axis variance) from -1.25 to +1.25.**
- **Work area dose result: the measurement of radiation doses of areas adjacent to the x-ray room. It is 0.28mSv. It is writing error. The dose result from the door to the toilet was from 0.09 to 0.28 (sorry! It was editorial error and still it was deviated in acceptable range)** **(Please see the revised table 3).**
- Table 4: It would be better to consider significant figures. I think showing figures down to the second decimal would be enough.

**Response: Okay, it is corrected accordingly. (Please see the revised table 4).**

**Reviewer #2:**

The paper presents a study of occupational exposure due to medical applications in Ethiopia. The paper is written in a clear and concise language. However, the paper should be revised before publication. The study rationale and design should be explained in more detail and the implications of the study should be summarized. (E.g., are there any measures taken to reduce doses/raise awareness for use of RPE?)

**Response: Thank you dear reviewer. The study rationale is explained from lines 100-117 of the revised track changed manuscript. The study design is cross-sectional study, i.e., both occupational radiation exposure dose and its associated factors are assessed at a point in time. Yes of course, training was given for radiology personnel about radiation effect, radiation protection, and proper use of RPE.**
