# Supplementary material for: Occupational radiation exposure dose and associated factors among radiology personnel in Eastern Amhara, Ethiopia
Source: PLoS One. 2023 May 25;18(5):e0286400. doi: 10.1371/journal.pone.0286400 (PMC10212102; doi:10.1371/journal.pone.0286400)
Supplement: Attachment — Submitted filename: Resopnse to Reviewers.doc [file pone.0286400.s004.doc]

**Response to Academic Editor**

Please review your reference list to ensure that it is complete and correct. If you have cited papers that have been retracted, please include the rationale for doing so in the manuscript text, or remove these references and replace them with relevant current references. Any changes to the reference list should be mentioned in the rebuttal letter that accompanies your revised manuscript. If you need to cite a retracted article, indicate the article’s retracted status in the References list and also include a citation and full reference for the retraction notice.

**Response:** Thank you so much. We tried to revise the references as per the journal requirement. We didn’t get retracted articles. Reference 2 is changed by the recent reference. Please see the track changed manuscript to see the specific changes made on the references list. Finally, the manuscript is revised considering both the academic editor and reviewer #1 comments.

**Response to Reviewer 1 Comments**

Reviewer #1: Almost all my previous comments seem to be considered by the authors. I appreciate the authors’ efforts for revising the manuscript. The only one point that I’d like to address again is the use of terminology. It is also commented from the academic editor.

According to your response shown below, there is no difference between “effective deep (or shallow) dose” and “deep (or shallow) dose”. If there is no difference between the two technical terms, I would suggest using “deep dose” and “shallow dose” to avoid confusion.

My previous comments

Lines 270-272, 275-286, 291, 292, 350-352, 364 and 368: effective deep and shallow doses

What are the definitions of “effective deep and shallow doses”? Are these different from “deep dose” and “shallow dose”? It should be clarified.

Your response

In case of TLD measurements and for our study, no difference. Effective deep dose or simply deep dose is a whole-body dose. Shallow dose or effective shallow dose (a shallow dose equivalent to the whole body) is a skin dose.

**Response:** Dear reviewer #1, thank you so much for the time you invested on the manuscript and the scientific ideas you suggested us to make the manuscript improved.

Regarding the terminologies “effective deep (or shallow) dose” and “deep (or shallow) dose”, in our study, since it is done on the whole body (not on a specific organ), deep dose and effective deep dose are the same. Similarly, shallow dose and effective shallow dose are the same. Therefore, as per your suggestion, the manuscript is corrected. Deep and shallow dose are used instead of effective deep and shallow dose.
