# Supplementary material for: Occupational radiation exposure dose and associated factors among radiology personnel in Eastern Amhara, Ethiopia
Source: PLoS One. 2023 May 25;18(5):e0286400. doi: 10.1371/journal.pone.0286400 (PMC10212102; doi:10.1371/journal.pone.0286400)
Supplement: Attachment — Submitted filename: Resopnse to Reviewers.doc [file pone.0286400.s005.doc]

**Response to Academic Editor**

**Journal Requirements:**

Please review your reference list to ensure that it is complete and correct. If you have cited papers that have been retracted, please include the rationale for doing so in the manuscript text, or remove these references and replace them with relevant current references. Any changes to the reference list should be mentioned in the rebuttal letter that accompanies your revised manuscript. If you need to cite a retracted article, indicate the article’s retracted status in the References list and also include a citation and full reference for the retraction notice.

**Response:** The reference list is complete and correct and we didn’t get retracted articles.

**Additional Editor Comments:**

This paper is much better in its present version. However, I still have some proposals for further improvements to make: Please use appropriate terminology on dose concept. The authors should check the definitions of the deep/shallow dose equivalent. Personal dose equivalent, Hp(10) and Hp(0.07) is an operational quantity for individual monitoring. Hp(10) is called the deep dose equivalent. Hp(0.07) is called the shallow dose equivalent. In addition, person-Sv is used for collective effective dose.

**Response:** Thank you so much for your concern. The terms deep dose and shallow dose are changed by deep dose equivalent and shallow dose equivalent. The definitions are also checked and it is correct. We used person-Sv instead of man-Sv for collective effective dose.
